# Xylitol production by *Saccharomyces cerevisiae* overexpressing different xylose reductases using non-detoxified hemicellulosic hydrolysate of corncob

**DOI:** 10.1007/s13205-016-0444-4

**Published:** 2016-06-07

**Authors:** Anushree Kogje, Anand Ghosalkar

**Affiliations:** 1 Department of Technology, Savitribai Phule Pune University, Pune, Maharashtra 411007 India; 2 Division of Praj Industries Limited, Praj-Matrix - R & D Centre, 402/403/1098, Urawade, Pune, Maharashtra 412115 India

**Keywords:** Xylitol, *Saccharomyces cerevisiae*, Xylose reductase, Corn cob hemicellulosic hydrolysate

## Abstract

Xylitol production was compared in fed batch fermentation by *Saccharomyces cerevisiae* strains overexpressing xylose reductase (XR) genes from *Candida tropicalis, Pichia stipitis, Neurospora crassa*, and an endogenous gene *GRE3.* The gene encoding a xylose specific transporter (*SUT1*) from *P. stipitis* was cloned to improve xylose transport and fed batch fermentation was used with glucose as a cosubstrate to regenerate NADPH. Xylitol yield was near theoretical for all the strains in fed batch fermentation. The highest volumetric (0.28 gL^−1^ h^−1^) and specific (34 mgg^−1^ h^−1^) xylitol productivities were obtained by the strain overexpressing *GRE3* gene, while the control strain showed 7.2 mgg^−1^ h^−1^ specific productivity. The recombinant strains carrying XR from *C. tropicalis, P. stipitis*, and *N. crassa* produced xylitol with lower specific productivity of 14.3, 6.8, and 6.3 mgg^−1^ h^−1^, respectively, than *GRE3* overexpressing strain. The glucose fed as cosubstrate was converted to biomass and ethanol, while xylose was only converted to xylitol. The efficiency of ethanol production was in the range of 38–45 % of the theoretical maximum for all the strains. Xylitol production from the non-detoxified corncob hemicellulosic hydrolysate by recombinant *S. cerevisiae* was reported for the first time. Xylitol productivity was found to be equivalent in the synthetic xylose as well as hemicellulosic hydrolysate-based media showing no inhibition on the *S. cerevisiae* due to the inhibitors present in the hydrolysate. A systematic evaluation of heterologous XRs and endogenous *GRE3* genes was performed, and the strain overexpressing the endogenous *GRE3* gene showed the best xylitol productivity.

## Introduction

Xylitol, the sugar alcohol is a promising polyol due to its application as a low calorie sweetener. It can be used for its antimicrobial properties to prevent dental and other infections (Nevoigt 2008). Xylitol is conventionally produced by hydrolysis and hydrogenation of xylan to xylitol under high temperature and pressure conditions. In spite of very high conversion efficiency, this technology has certain constrains viz. requirements of high pressure, temperature, expensive catalyst, and extensive downstream operations (Leathers 2003). Microorganisms convert xylose to xylitol by the cofactor-dependent xylose reductase (XR or *Xyl1*) enzyme (Saha 2003; Cocotle-Ronzon et al. 2012). The majority of essential cofactor NADPH/NADH for the XR activity is regenerated through pentose phosphate pathway (PPP) (Bengtsson et al. 2009; Schwartz et al. 2012). Several microorganisms have been screened and studied with respect to their ability for assimilation of xylose as carbon source and conversion to xylitol. Extensive research has been conducted to isolate and identify the suitable yeasts for xylitol production (Barbosa et al. 1988; Parajo et al. 1998a). Xylitol producing yeasts, such as *P. stipitis, C. tropicalis, C. guilliermondii*, *D. hansenii* (Mohamad et al. 2015), and *K. marxianus* (Zhang et al. 2013), have been studied for the optimization of the fermentation parameters, utilization of various nutrients, and fermentation of xylose rich hemicellulosic hydrolysate obtained from different sources of pretreated biomass (Parajo et al. 1998a, b; Dominguez et al. 1996; Ping et al. 2013). These organisms were preferred, as they exhibited efficient conversion of xylose to xylitol. Several reports on *Candida* species involved studies on the fermentation of pretreated biomass hydrolysates. Ghindea et al. (2010) have provided a review on various microorganisms studied for xylose transport and xylitol production.

*Saccharomyces cerevisiae* does not naturally utilize xylose as a carbon source, yet was preferred due to its GRAS (Generally Regarded As Safe) status. *S. cerevisiae* carries *GRE3* gene encoding a non-specific NADPH-dependent aldose reductase that converts xylose to xylitol (Kuhn et al. 1995). Many researchers have attempted to overexpress heterologous xylose reductases from *P. stipitis* or several *Candida* species in *S. cerevisiae* mainly for ethanol or xylitol production. However, strains overexpressing endogenous *GRE3* gene have not been evaluated systematically for xylitol production using media derived from pretreated biomass. The byproducts generated during the pretreatment of biomass, such as furans, weak acids, and phenolics, inhibit the cell metabolism individually or synergistically (Almeida et al. 2007; Hu et al. 2009). *S. cerevisiae* acquires tolerance to some inhibitors, such as HMF and furfural, due to the presence of some oxido-reductase enzymes (Heer et al. 2009).

The recombinant strains developed for xylitol production exhibit low productivity and xylose consumption rate in biomass derived media (Menon et al. 2010; Karhumaa et al. 2005). In the previous reports, *P. stipitis* XR has been cloned and overexpressed in *S. cerevisiae* for xylose metabolism. A detailed review on the various genetic engineering strategies of *S. cerevisiae* and fermentation of xylose containing media has been provided by Chu and Lee (2007). In some cases, multiple genes have been altered through or overexpressed, but endogenous gene expression levels have been rarely modified for xylitol production.

The present investigation deals with the evaluation of the conversion of xylose to xylitol by *S. cerevisiae* strains overexpressing different XR genes. The gene *SUT1* was cloned to avoid the limitation of xylose transportation in the *S. cerevisiae* cells. The xylitol production by the recombinant *S. cerevisiae* strains was compared in hemicellulosic hydrolysate of corn cob with the fed batch fermentation process.

## Materials and methods

### Cultures, growth media, and plasmids used

*Pichia stipitis* ATCC 58784*, Candida tropicalis* ATCC 9968, and *Neurospora crassa* NCIM 870 were used as a source of xylose reductase genes for which accession numbers have been provided in Table 1 (*PsXR, CtXR,* and *NcXR*, respectively). *GRE3* gene was amplified from *S. cerevisiae*, and *E. coli* TOP10F’ was used as intermediate host for the cloning and multiplication of the plasmid vector. The host strain used was *S. cerevisiae* BY4741. The yeast cultures of *P. stipitis*, *C. tropicalis*, and *S. cerevisiae* were grown and maintained in YPD media (10 g Yeast Extract, 20 g Peptone, 20 g Glucose per liter). *Neurospora crassa* was grown in FD medium containing per liter 5 g Peptone, 3 g Beef Extract, 1.5 g Potasium phosphate monobasic, and 1.5 g Potasium phosphate dibasic. Recombinant *S. cerevisiae* BY4741 cultures were grown in SD (Synthetic dropout) medium containing glucose 20 g and Yeast Nitrogen Base 6.7 g per liter supplemented with essential amino acids except uracil and leucine (Amberg et al. 2005). This selective medium was also used as prefermentation growth medium (PF). Luria–Bertani medium and ampicillin (80 ppm) were used for *E. coli* growth under selective pressure.

**Table 1:** Primers used in this study

Primer	Target gene (accession no.)	Primer sequence	Restrction Sites
*PsXR*-F	*P. stipitis* XR (NC_009045)	5′-GAC **GGA TCC** ATG CCT TCT ATT AAG TTG AAC-3′	*Bam*HI
*PsXR*-R		5′-TAA**CTCGAG**TTGTCCCAGTCCCATGGGTCGTTGAAT-3′	*Xho*I
*CtXR*-F	*C. tropicalis* XR (NW_003020040)	5′-GAGC**GGATCC**ATGTTTAAATTTTTCACTTCTCCAA-3′	*Bam*HI
*CtXR*-R		5′-CGCCA**CTCGAG**TTAAACAAAGATTGGAATGTTGT-3′	*Xho*I
*GRE3*-F	*S. cerevisiae GRE3* (NC_001140)	5′-CTC**GGATCC**ATGTCTTCACTGGTTACTCTT C-3′	*Bam*HI
*GRE3*-R		5′-ATA**CTCGAG**TCAGGCAAAAGTGGGGAATTTA-3′	*Xho*I
*NcXR*-F	*N. crassa* XR (NC_026501)	5′-CA**GAATTC**ATGGTTCCTGCTATCAAGCTCAA-3′	*Eco*RI
*NcXR*-R		5′-TCA**ATCGAT**CTAACCGAAAATCCAGAGGTTCT-3′	*Cla*I
*SUT1*-F	*P. stipitis SUT1* (NC_009068)	5′-TACACGTACTTAGTCGCTGAAGCTCTTCTATGATGTCTTCTCAAGATATTCCTTCAG-3′	NA
*SUT1*-R		5′-AGGTACGAACTCGATTGACGGCTCTTCTACCTTAAACATGTTCGTCAACAGGC-3′	NA

Restriction sites are indicated in bold

*NA* not applicable

The shuttle expression vector plasmid p426TEF (ATCC 87369) was used for cloning and episomal expression of the selected XR genes in *S. cerevisiae*. Cloning of the *P. stipitis SUT1* gene was done in the shuttle expression vector pD1211 (DNA 2.0 Inc., CA, US) as per the kit protocol. Both the vectors have 2µ origin of replication, high copy number, and a strong promoter TEF1 and CYC1 terminator flanking the multiple cloning site for high-level constitutive expression of the respective genes.

### Genetic manipulation of microorganisms

**Fig. 1:**
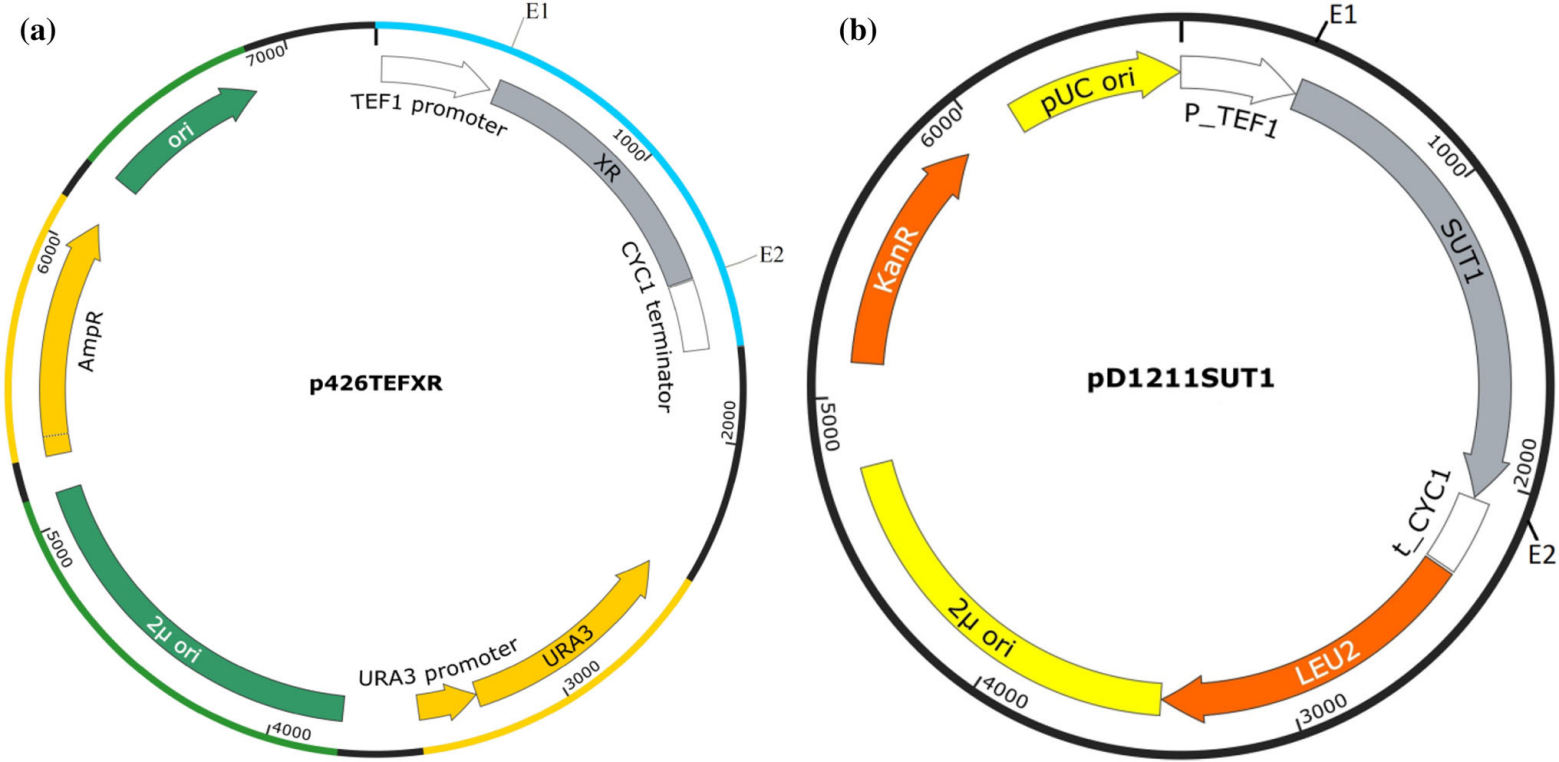
Plasmid map of **a** p426TEF with cloned XR, **b** pD1211 with *SUT1,* where E1 and E2—Restriction site in *forward* and *reverse* primers, respectively, used for cloning

The genes were PCR amplified from the genomic DNA of the source organism using the primers listed in Table 1. Genomic DNA preparation from yeasts or fungus was done by the Genomic DNA isolation kit Zymo-D6005 (Zymo Research). The plasmids and the genes were digested with the corresponding restriction enzymes, as indicated in Table 1, and purified by the Qiagen Gel Extraction kit. Ligation of the double digested gene and vector was done by the T4 DNA ligase. All the enzymes were purchased from NEB. Transformation of the *E. coli* cells was done by the standard heat shock method, and recombinant cells were selected on the plates of selective medium. Bacterial plasmid purification was performed with the Plasmid Mini kit 25 (Qiagen, Netherlands), and recombinant plasmids p426TEF carrying XR genes and the plasmid pD1211*SUT1* were co-transformed into yeast cells by Frozen-EZ Yeast transformation II kit (Zymo Research). The recombinant cells were selected on the SD media plates without uracil and leucine. Yeast plasmids were purified using Zymoprep Yeast Plasmid Miniprep II kit (Zymo Research) and used for verification. All the kits used for yeast samples were procured from Zymo Research, USA. The map for the plasmid carrying XR genes has been shown in Fig. 1. Transformed yeast colonies were screened and verified by PCR using gene specific primers. The confirmed colonies were grown in selective medium and stored as glycerol stocks for further use. The features of the plasmids used and the strains developed for this study are described in Tables 2 and 3, respectively.

**Table 2:** Plasmids used in the present study

Plasmid	Markers (bacterial, yeast)	Expression cassette (promoter, terminator)	Gene cloned
p426TEF	Amp^R^, Ura3	P_TEF_, T_CYC1_	–
pD1211	Amp^R^, Leu2	P_TEF_, T_CYC1_	–
p426TEF*PsXR*	Amp^R^, Ura3	P_TEF_, T_CYC1_	*PsXR*
p426TEF*CtXR*	Amp^R^, Ura3	P_TEF_, T_CYC1_	*CtXR*
p426TEF*NcXR*	Amp^R^, Ura3	P_TEF_, T_CYC1_	*NcXR*
p426TEF*GRE3*	Amp^R^, Ura3	P_TEF_, T_CYC1_	*GRE3*
pD1211*SUT1*	Amp^R^, Leu2	P_TEF_, T_CYC1_	*SUT1*

**Table 3:** Strains used in the present study

Strain	Description	Reference
*S. cerevisiae* BY4741	*MATa his3∆1 leu2∆0 met15∆0 ura3∆0* (ATCC No. 201388)	Brachmann et al. (1998)
ScpGT	BY4741 + p426TEF*GRE3* + pD1211*SUT1*	This study
ScpCT	BY4741 + p426TEF*CtXR* + pD1211*SUT1*	This study
ScpPT	BY4741 + p426TEF*PsXR* + pD1211*SUT1*	This study
ScpNT	BY4741 + p426TEF*NcXR* + pD1211*SUT1*	This study

### Preparation of the hemicellulosic hydrolysate of corn cob

Corn cob having total solid content of 92 % on the weight basis was subject to mechanical shear to obtain the particle size of 20–40 mm, which was then soaked in water for nearly 30 min. The feed containing 30 % solids was constantly supplied with steam, 2 % sulphuric acid, and 1 % oxalic acid on the basis of the solid content for pretreatment. The pretreatment was performed in the continuous digester for 20 min at high pressure and temperature of 6 bars and 160 °C, respectively, maintained using the acids and steam. The pretreated slurry of the hemicellulosic hydrolysate of corn cob thus generated contained about 18–20 % solids. The hemicellulosic hydrolysate of corn cob was used for fermentation trials, for which the composition is provided in Table 4.

**Table 4:** Composition of hemicellulosic hydrolysate of corn cob

Component	Concentration (gL^−1^)
Xylose	65
Glucose	7
Arabinose	6
Acetic acid	7
HMF + Furfueal	0.7
Phenolics	3–4

### Fermentation studies

The fermentation inoculum was prepared in two stages, where glycerol stock of the strains was used to start the first stage culture. The stage one culture (10 % vv^−1^) was then used to inoculate the stage two culture. In both stages of the inoculum preparation, cultures were grown in the PF medium at pH 5.5, 150 rpm and incubated at 30 °C for 24 to 30 h to achieve OD_600nm_ of about 15. Fermentation was started by inoculating 15 % vv^−1^ stage two inoculum to attain an initial OD_600nm_ of 2 in the fermentation broth. Concentrated stocks of YP (10X), glucose and xylose (50 gL^−1^ each), were sterilized separately, and corn cob hemicellulosic hydrolysate was heated at 80 °C for 10 min. The stock solution of was used for the fermentation trials with synthetic xylose, while the hemicellulosic hydrolysate was used as the source of xylose in corn cob hemicellulosic hydrolysate-based fermentation. The synthetic xylose stock or the hemicellulosic hydrolysate was added to YPD to obtain a final xylose concentration of 40 gL^−1^ in 400 ml of fermentation broth. Fermentation of xylose to xylitol by the recombinant strains overexpressing *SUT1* and an XR gene was performed by fed batch fermentation, as described by Chung et al. (2002), with some modification. Fed batch fermentation was carried out in the 1 L reactor (BioFlo/CelliGen 115, New Brunswick, USA) at temperature 30 °C, agitation of 150 rpm, and at 0.2 vvm aeration. Process was operated in batch mode for first 24 h after inoculation. Once the initial glucose was completely consumed, glucose stock solution (300 g/L) was continuously fed at a flow rate of 2.5 mL/h. The pH of medium was maintained at 5.5 using 4 N NaOH or 4 N HCl. Antifoam was added as per requirement. All the chemicals were purchased from HiMedia (India).

### Analysis of fermentation samples

The fermentation samples were analyzed for concentrations of glucose, xylose, xylitol, ethanol, and other byproducts by HPLC (1100 system, Agilent Technologies, USA). Separation was done using the Aminex HPX-87H column (300 × 7.8 mm i.d.) (Bio-Rad, USA) at a column temperature of 55 °C. Sulfuric acid (5 mM) was used as mobile phase at a flow rate of 0.6 mL/min, and the compounds were detected using the refractive index detector.

### Dry cell weight measurement

The fermentation samples were centrifuged at 8000 rpm for 5 min. The cell pellet was washed with distilled water. The cells were resuspended in 1 mL water and allowed to dry in a glass plate in the vacuum oven at 60 °C. The dry cell weight was calculated in g DCW per liter.

### Specific xylose reductase activity measurement

Fermentation samples were centrifuged at 8000 rpm for 5 min to harvest 50 mg cells and were washed with distilled water. Crude cell-free extract was prepared by treating cells with 200 µl Y-PER™ Yeast Protein Extraction Reagent (Thermo Scientific, USA) and 4 µl Protease Inhibitor Cocktail (Sigma Aldrich, USA) and then centrifugation at 13,000 rpm for 10 min. The supernatant or crude cell-free extract was immediately used for the enzyme assay and protein measurement or stored at −80 °C. The specific XR activity was estimated by the method reported earlier (Verduyn et al. 1985). The assay reaction contained 150 µM NADPH, 500 mM Potassium phosphate buffer having pH 6, 200 µM xylose, and diluted crude cell-free extract in a total assay volume of 1 mL. Reaction mixture was allowed to stand on ice for 10 min, and absorbance was read at 340 nm using the spectrophotometer (Schimadzu UV2450, Japan). One unit of XR activity is defined as the amount of enzyme required to oxidize one µM NADPH in 1 min. Protein content was determined by the standard method (Lowry et al. 1951). The specific XR activity was calculated by dividing the XR activity by the protein concentration in the samples.

### Mitotic stability measurement

The recombinant strains exhibited loss of vector during growth on non-selective media in fermentation studies. The stability of the recombinant strains was measured as the relative viability count. The fermentation broth was diluted appropriately and spread onto plates of selective (SD) and non-selective (YPD) media to estimate the fraction of recombinant cells that had lost the plasmid.

### Statistical analysis

The data were collected from at least two different and independent experiments. The statistical analysis was done using student’s *t* test null hypothesis and represented as mean ± standard error (S.E.).

## Results and discussion

### Genetic manipulation of microorganisms

Organisms carrying xylose reductase genes were identified on the basis of the high-specific XR activities viz. *P. stipitis, C. tropicalis*, and *N. crassa* based on the previous reports (Woodyer et al. 2005) along with the endogenous *GRE3* gene of *S. cerevisiae*. The gene *SUT1* from *P. stipitis,* encoding a xylose transporter was cloned along with XR genes in *S. cerevisiae* to increase the overall xylose metabolism. *Saccharomyces cerevisiae* does not carry an endogenous xylose transporter and it depends on the non-specific hexose sugar transporters (*HXT*) for xylose uptake (Hamacher et al. 2002). The xylose consumption rate of the *S. cerevisiae* strains engineered for xylose metabolism can be significantly improved by the expression of the *SUT1* gene (Katahiraa et al. 2008). Overexpression of *SUT1* gene was done to avoid any limitations of xylose uptake and to evaluate performance of different XRs cloned in the recombinant *S. cerevisiae*. Genetically engineered strains of *S. cerevisiae* were developed and verification was done by gene specific PCR.

### Fermentation using recombinant *S. cerevisiae* strains

**Fig. 2:**
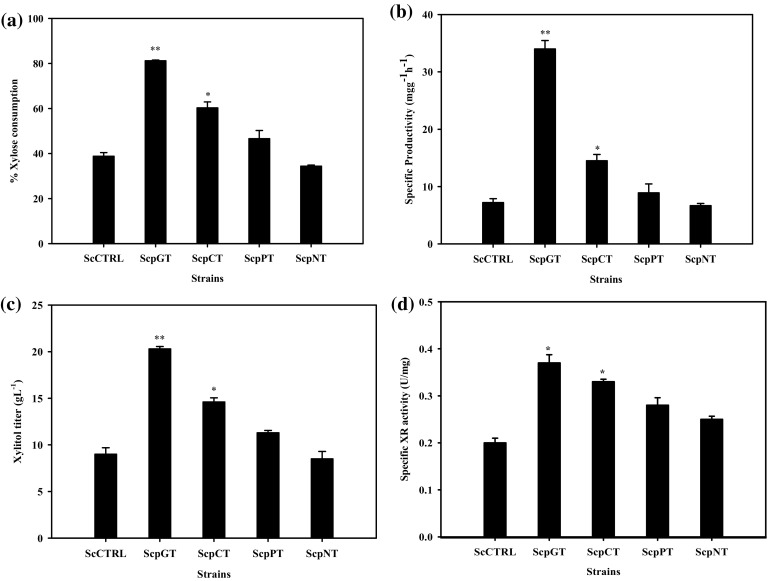
Percent xylose consumption **a**, specific xylitol productivity **b**, xylitol titer **c**, and specific xylose reductase activity **d** in fed batch fermentation with double recombinant strains overexpressing different *XR* and *SUT1* genes. *Difference* is indicated as significant (*p < 0.05, **p < 0.005) when compared to the control group

The functioning of xylose reductase is essentially dependent on cofactor NADPH, which could lead to a cofactor imbalance within cells. The fed batch fermentation process allowed the elimination of the redox imbalance arising due to the accumulation of NADP^+^ in the cell by maintaining carbon flux through PPP. Glucose limited fed batch fermentation process was adopted to achieve NADPH regeneration for the activity of xylose reductases essential for xylitol production, as shown in Fig. 2. The fermentation process was operated in batch mode for first 24 h to attain cell growth up to 9 gL^−1^ dry cell weight. Xylose consumption was not observed in the batch mode. Glucose feeding was done to allow continuous cofactor regeneration in the fed batch phase of xylose fermentation. Hector et al. (2011) showed that NADPH is regenerated mainly through PPP during glucose metabolism. The glucose feed rate of 0.75 gh^−1^ was maintained which was just enough for the growth and did not allow its accumulation in the broth to avoid catabolite repression of xylose consumption in the recombinant *S. cerevisiae*. Xylitol productivities of the recombinant strains were compared with the control. All the recombinant and the control strains showed nearly theoretical yield of xylitol on consumed xylose.

Xylitol production rate by strains containing different xylose reductases has been summarized in Fig. 2a–d. The control *S. cerevisiae* strain carrying empty plasmid consumed about 38.8 % of the initial xylose and produced xylitol at a near theoretical yield indicating no further xylitol metabolism. Figure 2a shows that the strain overexpressing *GRE3* gene showed 81 % consumption of the initial amount of xylose, and it produced xylitol with a specific productivity of 34 mgg^−1^ h^−1^, which was about fivefold higher than the control strain. The strain overexpressing XR from *C. tropicalis* also showed a significant increase of twofold over the control strain. Overexpression of the *Pichia* and *Neurospora* XR showed specific xylitol productivity of 9.8 and 6.6 mgg^−1^ h^−1^, respectively, which was not a significant increase over the control strain (7.9 mgg^−1^ h^−1^), as shown in Fig. 2b. The final titer of xylitol obtained during fermentation by different strains is shown in Fig. 2c. The results indicated that the strain overexpressing *GRE3* gene produced the highest xylitol up to 21 gL^−1^ followed by the strain carrying *C. tropicalis* XR with 15 gL^−1^. The other recombinant strains produced xylitol at similar titers to the control strain. The highest amount of xylitol obtained in the broth by the strain carrying *GRE3* gene was twofold higher than the control strain.

Comparison of the recombinant strains overexpressing *XR* and *SUT1* showed that increase in the specific enzyme activity of recombinant xylose reductase enzyme in *S. cerevisiae* showed a corresponding increase in xylitol production by the recombinant strains. The biochemical characteristics of different XRs in their native hosts have been summarized in Table 5 (Woodyer et al. 2005). There was a difference between the catalytic efficiency of these reductases in their native hosts and their activities in *S. cerevisiae* observed in this study (Table 5). In the case of the catalytic activities in their native hosts, the XR from *Neurospora* showed the highest turnover number (*K*_cat_) with xylose and NADPH as substrates.

**Table 5:** Properties of xylose reductases in their native systems (Woodyer et al. 2005)

Organism	Enzyme Mol wt (kDa)	*K*_m_ for xylose (mM^−1^ min^−1^)	K_cat_ (min^−1^)
*S. cerevisiae*	33	13.6	860
*C. tropicalis*	58	35	ND
*P. stipitis*	65	42	1500
*N. crassa*	53	34	3600

**Table 6:** Ethanol production by the recombinant *S. cerevisiae* strains

Strain	Ethanol titer (gL^−1^)	*Y*_p/s_ (gg^−1^)^a^	Efficiency (%)^b^
Sc RT	23.36	0.22	44.04
Sc CT	22.81	0.22	43.00
Sc PT	21.83	0.21	41.17
Sc NT	23.87	0.23	45.01
Sc control	20.32	0.20	38.31

^a^ Yield of ethanol produced on glucose consumed

^b^ Percent efficiency of the yield obtained and the theoretical maximum of ethanol on glucose

Xylose metabolism by *S. cerevisiae* has been attributed to the endogenous non-specific NADPH-dependent aldo-ketoreductase enzyme encoded by the gene *GRE3*. This gene is reported to be expressed in stress-related responses, such as hypoxia, heat shock, starvation, heavy metals, osmotic, and ionic stresses, including lithium (Celton et al. 2012; Garay-Arroyo and Covarrubias 1999). Its sequence analysis shows close relation with the reductases from the xylose fermenting yeasts (Kuhn et al. 1995).

**Fig. 3:**
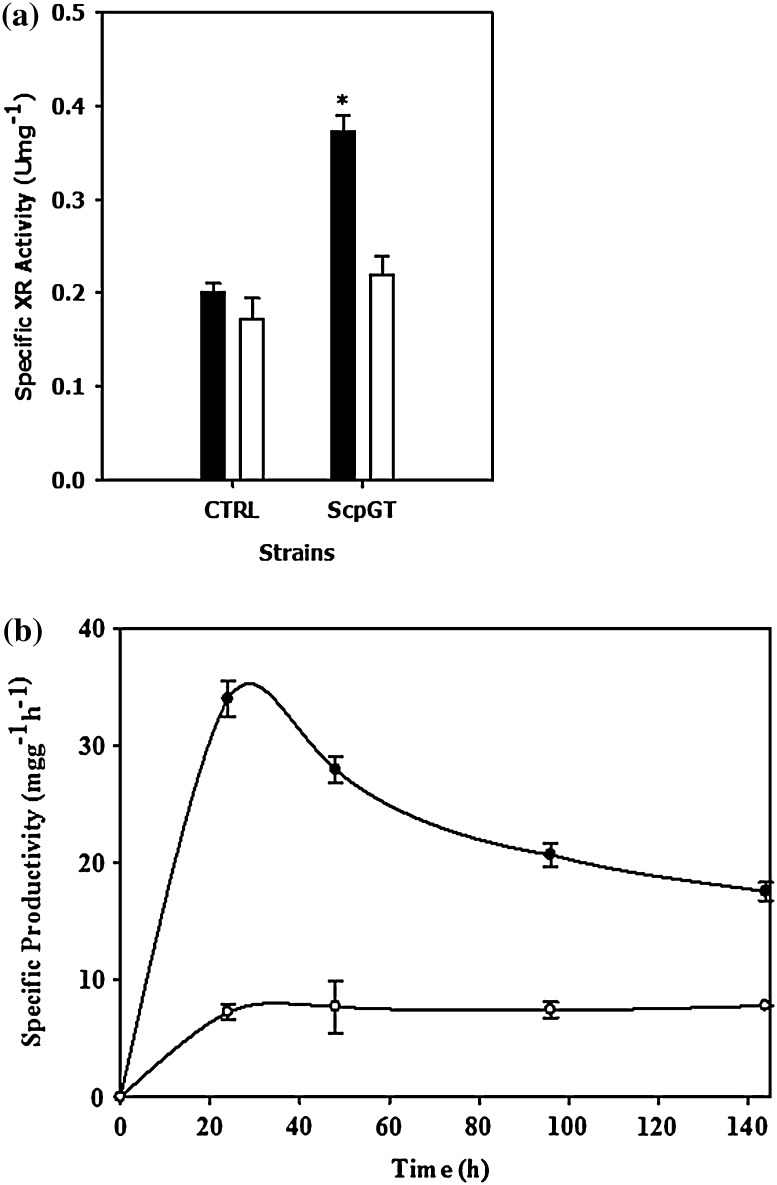
Mitotic instability of recombinant strain leads to decrease in xylitol productivity **a** specific XR activity of Sc pGT (*dark fill*) and control (*no fill*) and **b** specific productivity of *S. cerevisiae* overexpressing *GRE3* gene ScpGT (*solid symbols*) and control (*empty symbols*) over time. Values are expressed as Mean ± S.E. (*n* = 2)

The strain carrying *GRE3* was observed to have the highest specific XR activity followed by the strain carrying *CtXR* over the control strain (Fig. 2d). The specific xylose consumption rate and xylitol productivity both were found to be significantly higher in the strain carrying *GRE3* gene followed by the strain carrying *CtXR* gene around 34 and 28 mgg^−1^ h^−1^, respectively. The highest specific XR activity (Fig. 3a) and xylitol productivity (Fig. 3b) were observed in the early phase of fermentation which decreased at later time points. Expression of the genes *PsXR* and *NcXR* did not lead to a significant improvement in the xylitol productivity over the control, as shown in Fig. 2. This could be attributed to the higher *K*_m_ values for xylose of these enzymes (34, 42 mM, respectively) as compared with the product of the gene *GRE3* (13.6 mM) (Woodyer et al. 2005). *NcXR* has been reported to enhance the fermentation of xylose when cloned in xylitol dehydrogenase (XDH) deleted strain of *C. tropicalis* (Jeon et al. 2012). However, the overall consumption of xylose and xylitol productivity of the *S. cerevisiae* strain carrying *NcXR* was not significantly higher than the control strain (Fig. 2). The relative differences in the specific xylitol productivity (Fig. 2b) of different strains over time showed that the overall fermentation efficiency was dependent on the efficiency of the enzyme as well as the mitotic stability of the cells over the same amount of biomass in the broth. These observations highlighted that the production of xylitol was only regulated by the efficiency of the reductase enzyme during fed batch fermentation. This limitation was overcome by overexpression of the appropriate gene in these strains along with the xylose transporter.

**Fig. 4:**
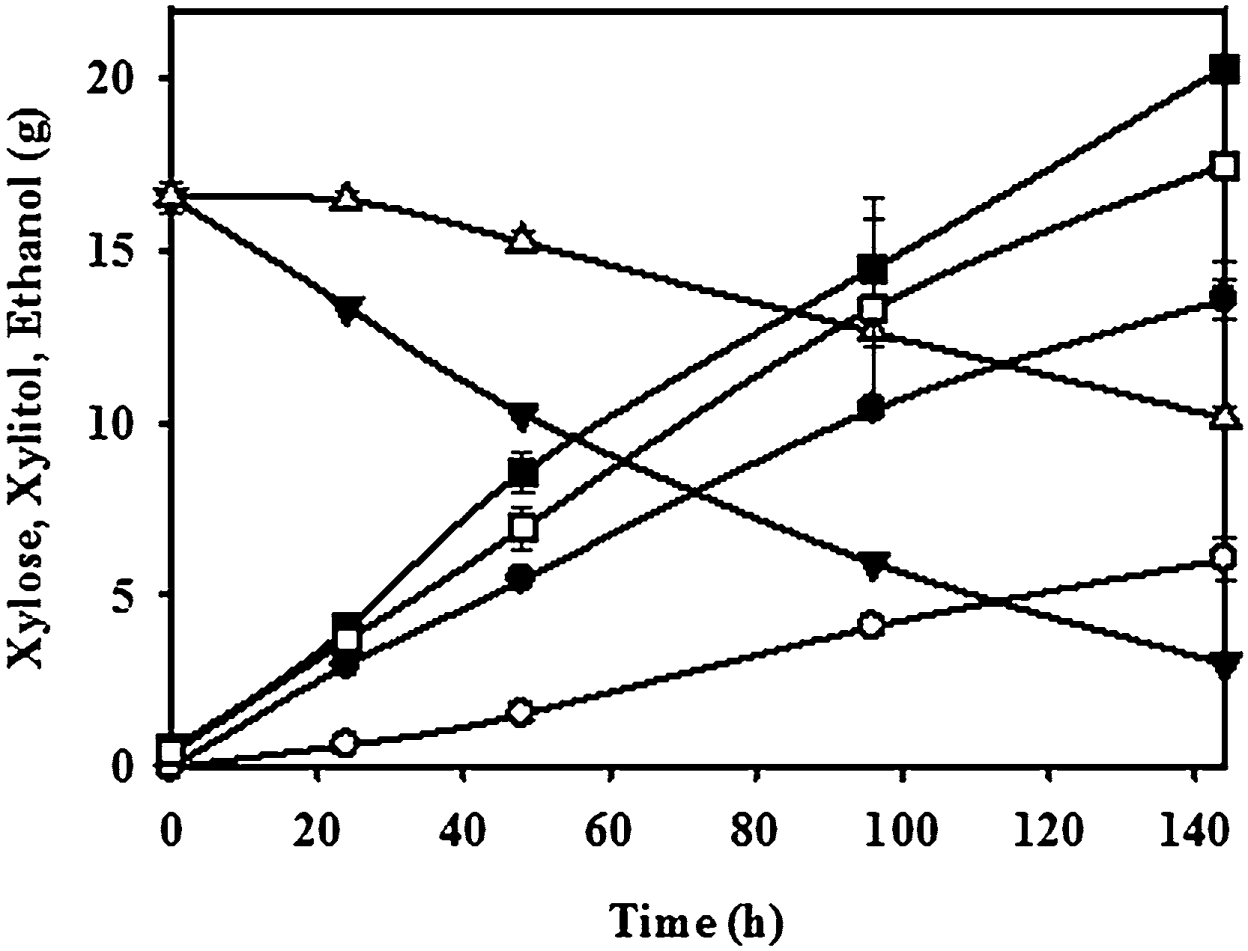
Fermentation profiles of control (*empty symbols*) and ScpGT (*filled symbols*) strains, where Xylitol—c*ircle*, Xylose—*triangle*, and Ethanol—*square*. Values are expressed as Mean ± S.E. (*n* = 2). *Asterisk* indicates a significant difference (*p* < 0.005) when compared to the control group

Ethanol was produced from the glucose consumed during fed batch fermentation, and xylose was converted to only xylitol, as the gene coding for the enzyme xylitol dehydrogenase is not present in *S. cerevisiae* to further metabolise xylitol. The efficiency of ethanol production was determined as the ratio of the yield of ethanol obtained to the theoretical maximum ethanol yield (0.51 g per g glucose). All the recombinant *S. cerevisiae* strains showed ethanol production efficiency in the range of 38–45 % from the glucose during fed batch fermentation (Table 6). The profiles of the xylose consumed, xylitol produced along with the amount of ethanol formed over time by the control, and *GRE3* overexpressing strains have been shown in Fig. 4. The figure shows that the rate of xylose consumption by the strain overexpressing *GRE3* gene was higher than the control strain, while the accumulation of ethanol followed almost similar trend in both strains indicating a constant rate of glucose metabolism without accumulating it in the broth.

**Table 7:** Fed batch fermentation with *S. cerevisiae* overexpressing *GRE3* with *SUT1* in synthetic xylose and hemicelullosic hydrolysate of corn cob (HHCC) was comparable

Xylose consumed (%)		Titer (gL^−1^)	
HHCC	Synthetic	HHCC	Synthetic
84.4	80.9	22.4	19.7

We compared the xylose fermentation efficiency of the recombinant *S. cerevisiae* strains in media containing synthetic xylose as well as in non-detoxified hemicellulosic hydrolysate of corn cob. It was observed that there was no significant difference in the fermentation efficiencies in two media, as shown in Table 7. The comparison of the fermentation efficiencies in both the media confirmed that *S. cerevisiae* is the most suitable host for the processes involving industrially relevant media. The organisms belonging to the genus *Candida* have been extensively reported for high xylitol productivities (Ko et al. 2006). However, the reported yield of xylitol in fermentation of hemicellulosic hydrolysate is lower than that in the pure substrate (Walther et al. 2001; Choi et al. 2000). This is mainly due to the presence of inhibitory compounds arising from the pretreatment of biomass and the loss of xylose for cell growth or ethanol produced. As given in Table 4, the media used in this study contained the inhibitory compounds furfural, HMF, phenolics, and acetic acid at concentrations which are otherwise inhibitory to the metabolism and growth of the microorganisms, such as *C. tropicalis*. *S. cerevisiae* demonstrates a natural tolerance against such fermentation and metabolic inhibitors by the virtue of elevated function of the PPP and oxido-reductase enzymes giving it an advantage over other organisms (Celton et al. 2012).

### Specific XR activity estimation in fermentation samples

The maximum specific XR activities were attained by all the recombinant strains at the start of the xylose fermentation phase when residual glucose was not observed in the broth. The specific XR activity in the strain overexpressing *GRE3* gene was found to be 0.37 Umg^−1^ which was a significant improvement of 1.9 fold over the control strain. The strains carrying *CtXR*, *PsXR*, and *NcXR* genes showed 1.7, 1.5, and 1.3 fold specific XR activities, respectively, as compared to the control, as shown in Fig. 2d. Under the same host and vector system, *GRE3* gene showed the highest activity in *S. cerevisiae* resulting into highest productivity of xylitol.

Other studies on overexpression of *GRE3* along with different dehydrogenases and xylulose kinase (*XKS1*) genes in *S. cerevisiae* are focused on the improved fermentation of xylose to ethanol (Konishi et al. 2015; Khattab and Kodaki 2014). Similar to our findings, the authors have reported that the overexpression of *GRE3* gene leads to higher xylose fermentation. However, these studies are limited to the comparison of the fermentation efficiency of *S. cerevisiae* strains carrying *GRE3* and *XR* from *P. stipitis* for ethanol production. In another report on xylitol production by the *S. cerevisiae,* the efficiency of the strains expressing *GRE3* and *XR* from *P. stipitis* was compared (Kim et al. 2002). The authors have reported that in spite of the similar amounts of enzyme expression, the strain expressing *GRE3* showed lower xylitol productivity due to its strong preference to NADPH, unlike *P. stipitis XR* which also accepted NADH as cofactor. In our study, continuous glucose feeding strategy ensured a sufficient regeneration of NADPH for the activity of xylose reductase. We have found that under the same expression system, the enzyme activity of *CtXR* was also higher than *PsXR* leading to a higher xylose consumption rate.

### Effect of mitotic instability during fermentation

Since the expression system was a plasmid-based episomal system, loss of plasmid by cells was observed in the absence of selection pressure. This eventually led to the decrease in XR activity with progress of fermentation. It was observed that only up to 50 % of cells could stably retain plasmids at the end of fermentation. The effect of plasmid loss on the specific XR activity and xylitol productivity is shown in Fig. 3. The strain overexpressing *GRE3* gene showed about 50 % decrease in the reductase activity (Fig. 3a) and specific productivity of xylitol (Fig. 3b). However, the specific xylitol productivity of the control strain was constant throughout fermentation, since the enzyme activity of the genome encoded *GRE3* gene was constant with respect to the biomass, as shown in Fig. 3a.

## Conclusion

This is the first report on xylitol production using non-detoxified hemicellulosic hydrolysate of corn cob by recombinant *S. cerevisiae.* As a GRAS organism, *S. cerevisiae* is a preferable biocatalyst with high tolerance to inhibitory compounds present in hemicellulosic hydrolysates. It converts xylose to xylitol with near theoretical yield, whereas *Candida* sp. also utilizes it for cell growth. In the present study, a systematic evaluation of different reductase genes was done in recombinant *S. cerevisiae* containing heterologous xylose transporter. The *s*train overexpressing the endogenous *GRE3* gene along with the xylose transporter gene *SUT1* showed the highest xylitol productivity over control as compared to the strains carrying *C. tropicalis, P. stipitis*, and *N. crassa* xylose reductases. The fed batch process involved feeding of cosubstrate glucose, which could be available in the bio-refineries based on cellulosic substrates. Ethanol produced from the glucose fed during process can be recovered from the fermentation broth to improve the overall process economics. It can also help in the prevention of contamination during fermentation in the hydrolysate-based media. Fermentation was conducted under microaerobic conditions which was lesser than the aeration required for xylitol production by other naturally xylose fermenting yeast species, such as *Candida*.
